# Ultrasound prediction of abnormal infant development in hypertensive pregnant women in the second and third trimester

**DOI:** 10.1038/srep40429

**Published:** 2017-01-16

**Authors:** Guofang Shen, Yajuan Huang, Lixin Jiang, Jinghong Gu, Yaxin Wang, Bing Hu

**Affiliations:** 1 Department of Ultrasound in Medicine, Shanghai 6th People’s Hospital affiliated to Shanghai Jiaotong University, No 600 Yishan Road, Shanghai 200233, PR China; 2 Department of Obstetrics and Gynecology, Shanghai 6th People’s Hospital affiliated to Shanghai Jiaotong University, No 600 Yishan Road, Shanghai 200233, PR China

## Abstract

The objective was to assess the sensitivities and accuracies of Doppler ultrasound parameters in the second and third trimester of hypertensive pregnancies in determining perinatal outcomes. 1,054 pregnancies were retrospectively categorized into three groups (healthy pregnancies (HP, n = 988), pregnancies of hypertensive women (HypP, n = 30) and high-risk hypertension pregnancies (HRHypP, n = 36), depending on gestational hypertension as well as fetal birth weights and pregnancy outcomes. Systolic/diastolic ratio (S/D), resistance index (RI), pulsatility index (PI) of the bilateral uterine artery, umbilical artery and vein as well as venous flow velocity data were monitored by Doppler ultrasound. At 20–27 and 28–32 gestational weeks, uterine artery PIs and RIs were significantly higher in the HRHypP group than in the HP and HypP patients. At gestational weeks 20–27 and 28–32 left plus right PI data with cut-off values of 2.35 and 1.73 indicated a risk of stillbirth, premature pregnancy termination and a birth weight of less than 2,500 g with sensitivities of 94.4% and 93.1% as well as specificities of 95.2% and 90.1%, respectively.

Gestational hypertension (GH) is a high-risk factor for serious pregnancy outcomes, with a previously reported incidence of 5.22% for all pregnancies in China^1^. In another report, 10% of women had high blood pressure during pregnancy worldwide, with pre-eclampsia complicating 2% to 8% of pregnancies^2^. A complication of hypertension during pregnancy is intrauterine growth restriction/retardation (IUGR) due to a reduced blood supply to the fetus, which is associated with abnormal perinatal outcomes including preterm birth and perinatal morbidity^3^,^4^. In an earlier study of complicated pregnancies, correlations were found between abnormal perinatal outcomes and abnormalities of the uterine artery blood velocity waveform. The predictive value for an abnormal outcome was highest for an early diastolic notch followed by an increased mean uterine arterial PI and blood velocity on the placental side^5^. Siddiqui *et al*.^6^ found that 90% of IUGR cases with abnormal umbilical artery Doppler velocimetry waveforms had poor perinatal outcomes including early delivery, Cesarean section, and prenatal and neonatal death compared to 33% who retained normal Doppler flow^6^. Alfirevic *et al*.^7^ analyzed data from 18 studies involving 10,156 women and concluded that current evidence suggests a reduced risk of perinatal deaths and less obstetric interventions in high-risk pregnancies by using Doppler ultrasound. Doppler studies of the umbilical artery should be carried out for fetal monitoring, particularly in high-risk pregnancies with hypertensive disorders^7^. However, the authors stressed that the evidence was not of high quality and in a previous study they stated that the use of routine umbilical artery Doppler ultrasound in low-risk or unselected populations would not benefit the mother or the baby^8^.

The bilateral uterine artery is the main blood supply to the placenta, while the umbilical artery and vein are the main blood vessels in the placenta and fetus. The aim of this study was to observe and compare any differences between umbilical and uterine blood flows detected by Doppler ultrasound in healthy and gestational hypertension pregnancies. We hypothesized, that particularly a hypertension related uterine arterial blood flow reduction might be a factor for unfortunate pregnancy outcomes. We determined the Doppler ultrasound threshold pattern for estimating fetal wellness in the second and third trimester of pregnancy.

## Patients and Methods

**Figure 1:**
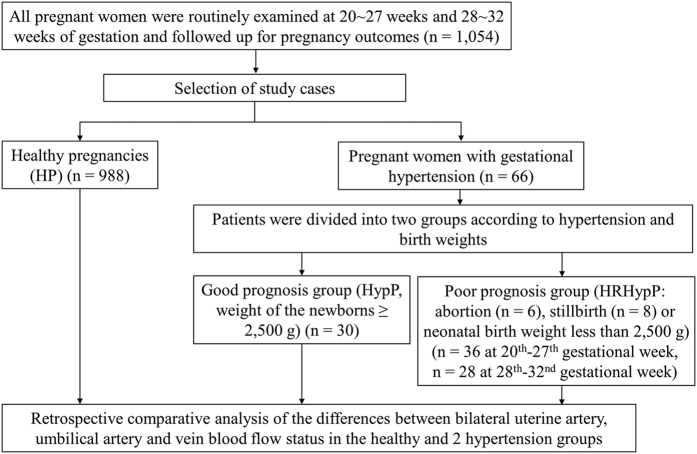
Flow chart of the study.

This was a retrospective study to identify potential ultrasound-markers of pregnancy complications in hypertensive pregnant women. The study, which was approved by Shanghai Sixth Hospital Research Ethics Committees, was carried out in the Shanghai Sixth Hospital in Jiaotong University and included patients admitted to the Shanghai Sixth Hospital from April 2013 to May 2015, who gave birth. The study was carried out in accordance with the approved guidelines and written informed consents were obtained from all subjects included in the study. In the Shanghai Sixth Hospital, all pregnant women routinely receive examinations at 20–27 and 28–32 weeks of gestation in order to confirm the gestational age from the measurement of the fetal crown-rump length and last menstrual period, to diagnose any major fetal abnormalities and monitor fetal growth and development. In our study, Doppler ultrasound data from 1,054 pregnant women including healthy pregnancies (HP) (n = 988), pregnancies with hypertension (HypP) (n = 30) and 36 pregnancies with high-risk hypertension (HRHypP) (n = 36) were retrospectively analyzed. Inclusion criteria for the HP group were: age ≥18 years and <35 years; no hypertension; no heart disease; no diabetes; no chronic nephritis or other diseases, and a fetal birth weight ≥2,500 g and <4,000 g; for the HypP group, patients were selected with hypertension and fetal birth weight ≥2,500 g, whereas the HRHypP group comprised pregnant women with hypertension and fetal birth weight <2,500 g (including stillbirths and abortions) (Fig. 1). Hypertension was defined as continuous systolic pressure >140 mmHg and diastolic pressure >90 mmHg.

### Uterine artery and umbilical blood flow detection

Ideally, measurements have to be carried out with several consecutive identical waveforms, with the sonation angle as close to zero as possible (Burns 1993). Data from umbilical artery, umbilical vein, as well as left and right uterine arteries were monitored. After pregnant women rested for 15 minutes, they lay down in the prone position or with a 15-degree turn onto their left side. First, we verified the fetal position and located the fetal ventral surface and body, after which we monitored the left and right uterine blood flows at 1 cm above the crossing point of the external iliac artery and both sides of the uterine artery, as well as the umbilical artery and umbilical vein on the free-end of the umbilical cord, with an ultrasonic probe (Voluson E8 S6 color Doppler ultrasound diagnostic instruments, GE Healthcare, Fairfield, US) at an abdominal probe frequency of 3.5 ~ 5 MHz; 3–5 continuous stable images of the standard waveforms were taken. The built-in software calculated the S/D ratio, RI, PI and venous flow velocity automatically.

### Statistical analysis

All analyses were performed using IBM SPSS Statistics for Windows (Version 19.0. Armonk, NY: IBM Corp.) A sample size of 988 cases in total was considered to provide the assumed significant level of 0.05 (2-sided); with a detection specificity and sensitivity of 90% and permissible deviation of 0.1, with an expected HRHypP incidence rate of 3.5% as well as accounting for a 5% dropout; 1,040 cases were included. The qualitative data were described by frequency (%) and the quantitative indicators were described as the mean ± standard deviation. Qualitative data were analyzed by chi-square or Fisher exact probability tests. Quantitative data between the two groups were compared using a *t*-test; comparison between multiple groups was carried out using analysis of variance. Multiple groups with statistically significant differences were compared by the SNK method. Sensitivity, specificity and likelihood ratios (LR) and + LR, as well as 95% confidence intervals (CI) for detecting HRHypP were calculated. We have also documented the inter observer reliability (two observers), intra-class correlation coefficients (ICC). ROC curve analysis was used to determine the optimal critical value of HRHypP diagnosis and the area under the curve was calculated.

## Results

### Basic characteristics of enrolled pregnant women

In the HypP and HRHypP groups, maternal age, previous history of PE, preterm birth rates, in addition to mean arterial BP and history of hypertension before pregnancy were all significantly higher than in the HP group. The maternal age and IUGR rates in the HRHypP patients were also significant higher and the fetal birth weight as well as fetal length was significantly lower than in the HypP group, but there was no difference between the HypP and HP women (Table 1).

### Ultrasound parameter changes in the uterine arteries as well as umbilical artery and vein in the 3 different pregnancy groups during the 20^th^ to 27^th^ gestational weeks

**Figure 2:**
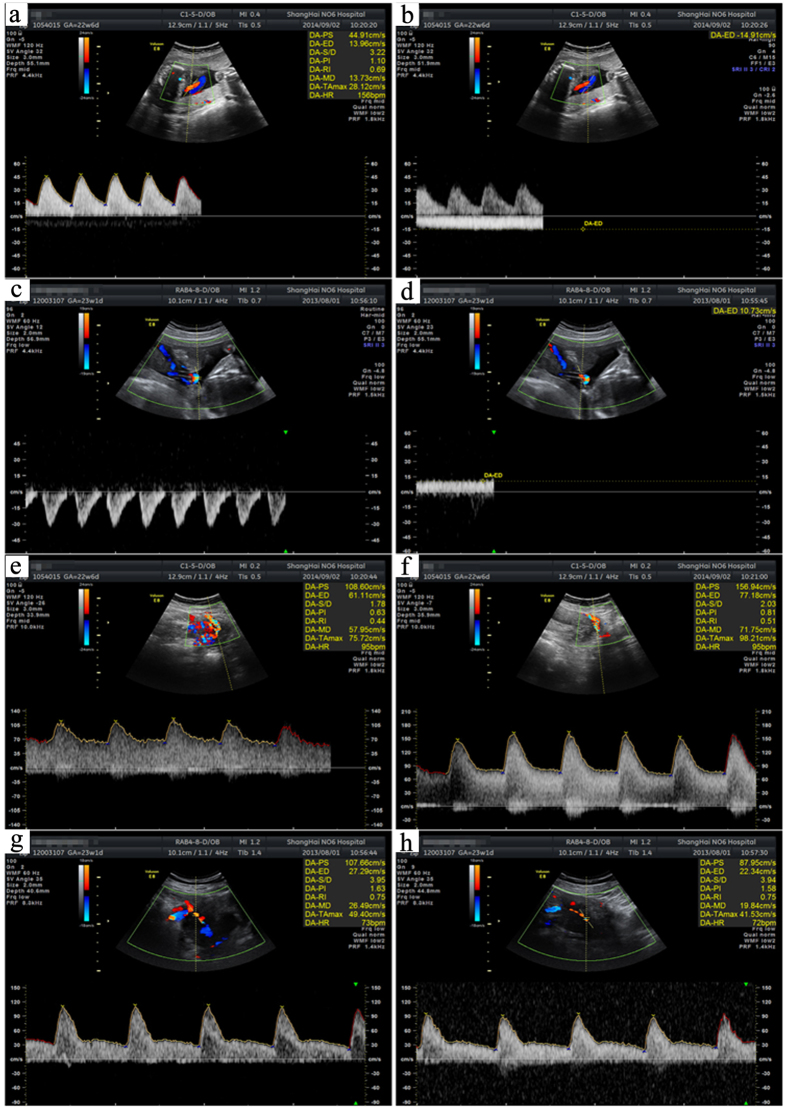
Representative Doppler ultrasound images at ~23 weeks of pregnancy. (**a**) Normal pregnancy umbilical artery. (**b**) Normal pregnancy umbilical vein. (**c**) HRHypP (umbilical artery with fetal death. (**d**) HRHypP umbilical vein with fetal death. (**e**) Normal pregnancy right uterine artery. (**f**) Normal pregnancy left uterine artery. (**g**) HRHypP right uterine artery with fetal death. (**h**) HRHypP uterine artery with fetal death.

There was no significant difference between the indicated indexes in the HypP and HP groups, but the HRHypP group exhibited significantly higher values except for umbilical vein flow velocity, when the values were significantly lower (Table 2, Fig. 2).

### Ultrasound parameter changes in the uterine arteries as well as umbilical artery and vein in the 3 different pregnancy groups during the 28^th^ to 32^nd^ gestatinal weeks

As in gestational weeks 20–27, there was no significant difference between the various indexes in the HypP and HP groups, but the HRHypP group exhibited significantly higher values except for umbilical vein flow velocity, where the values were significantly lower also in the 28^th^ to 32^nd^ gestational week (Table 3).

### Doppler ultrasound pattern analysis for the prediction of poor perinatal outcomes

**Figure 3:**
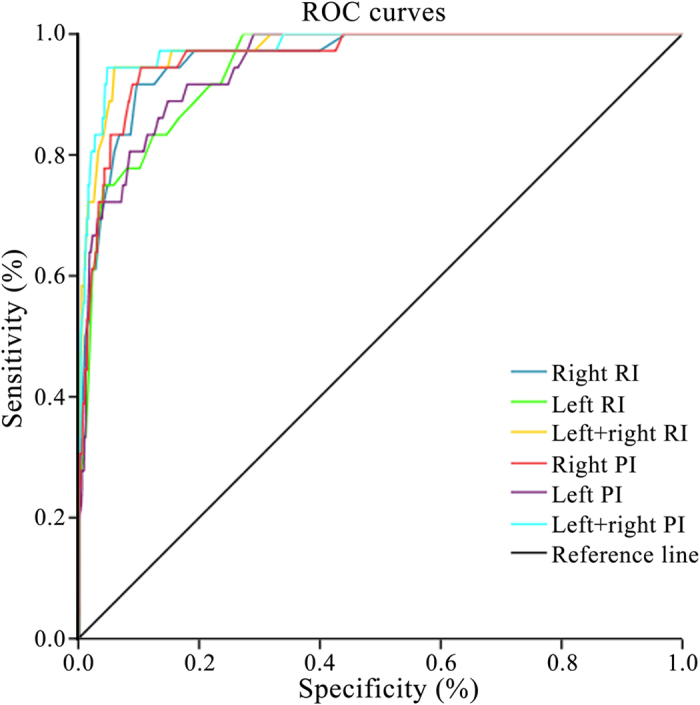
Sensitivity and specificity of ultrasound prediction indices in gestational weeks 20–27 in ROC curves.

**Figure 4:**
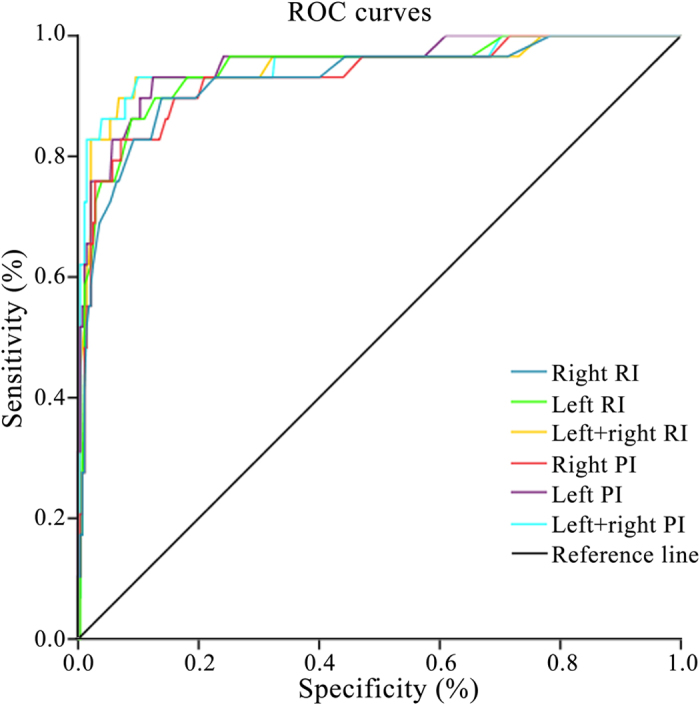
Sensitivity and specificity of ultrasound prediction indices in gestational weeks 28–32 in ROC curves.

Next, we used the data of 20–27 weeks after gestation to analyze the factors in HRHypP pregnancies for early ultrasound prediction of poor perinatal outcomes. Sensitivity and specificity of ultrasound prediction indices, derived from uterine arteries of the HRHypP group, were calculated and the sensitivity and specificity of left + right RI were found to be 88.9% and 94.5%, with a cut-off value of 1.26 in the 20^th^–27^th^ gestational weeks. The cut-off value of left + right PI was 2.35 with a sensitivity of 94.4% and the specificity was 95.2%. Similarly, in gestational weeks 28–32, the sensitivity and specificity of left + right RI were found to be 93.1% and 90.4%, with a cut-off value of 1.07. The cut-off value of left + right PI was 1.73 with a sensitivity of 93.1% and a specificity of 90.1%. (Table 4, Figs 3 and 4).

In order to determine the intra-and inter observer reliability for the PI/RI/Vmax measurements, we calculated the intra-class correlation coefficients (ICC) and 95% confidence intervals (CIs) (Table 5).

## Discussion

In the present study, we used combined left + right uterine artery PI/RI values to assess the prognosis and susceptibility of the fetus and found that abnormal left and right uterine artery PI values were correlated with a higher risk of stillbirth, premature pregnancy termination and a birth weight of less than 2,500 g, which is in agreement with previous reports^9^,^10^,^11^,^12^,^13^. The mean uterine artery PIs of healthy pregnant women in our study were 0.77 ± 0.23 and 0.66 ± 0.21 at 20–27 and 28–32 pregnancy weeks, whereas in HRHypPs they were 1.48 ± 0.35 and 1.41 ± 0.48, respectively (Table 6). There was no difference between the HP and HypP groups.

Our data are similar to previously published PI values of 0.80–0.73 in healthy women vs 1.47–1.43 in pre-eclampsia/gestational hypertension patients between 28 and 32 gestational weeks in high altitudes^14^, and 1.42 ± 0.23 in women with persistent hypertension between 26–28 gestational weeks^15^.

However, there was no statistically significant uterine atery PI difference between healthy and hypertensive pregnant women with good prognoses (Tables 2 and 3). Also, the fetal birthweight and fetal length did not differ between the hypertension and healthy pregnancies, though the preterm birthrate was higher in the hypertension group (Table 1). This data indicates that hypertension during pregnancy is not *per se* a factor for unfavorable outcomes and that PI values are not automatically abnormal in hypertensive pregnant women, findings reported in a previous study^16^. In addition, in the late-onset of gestational hypertension, abnormal uterine artery resistance is less harmful than in the early-onset cases^17^. Our PI cut-off value of >2.35 for both uterine ateries (>1.175 for one) in the 20^th^ to 27^th^ week of gestation is in the range of ≥1 proposed by Hafner *et al*.^18^ for PI values at the 22^nd^ gestational week^18^. Several authors noted that abnormal PI values are indicators of higher rates of maternal and fetal diseases, but false positive rates were also high. With a 10% false-positive rate, early-onset PE could be predicted in 70.6% of pregnancies, and subsequent IUGR in 73.3% of pregnancies with uterine artery Doppler PIs, and the authors proposed that especially for late onset PE maternal risk factors, it should also be taken into consideration^19^. Also, Papageorghiou *et al*.^20^ suggested that beside PI data, significant independent factors for predicting pre-eclampsia were dependent on ethnic origin, BMI, parity, cigarette smoking, history of hypertension and a family or personal history of pre-eclampsia. In the latter study, for a false-positive rate of 25%, the PE detection rate with uterine artery Doppler was 63.1% but using maternal history alone it was 45.3%, and with a combination of both parameters the detection rate was 67.5%^20^. Also in our study, 6.7~11.1% of the hypertensive group patients had a history of preeclampsia and 16.7% a history of hypertension, and the age of the HRHypP group was significantly higher than in the other groups, which might also be considered to be risk factors for unfortunate pregnancy outcomes.

A major clinical concern is that false positive Doppler ultrasound findings could encourage inappropriate early delivery, but a previous meta-analysis that included 10,000 patients showed that the use of Doppler ultrasound in high-risk pregnancies was associated with reductions in perinatal deaths, fewer inductions of labor and fewer Cesarean sections. However, the authors pointed out that the current evidence was not of high quality and that the results should be interpreted with caution^7^. A limitation of our study was the relatively small number of gestational hypertension patients and generalizability of the results will need external validation with a larger cohort of patients.

## Conclusion

In this retrospective study, we found that hypertension in combination with a cut-off value of the left and right uterine artery PI of >2.35 at the 20^th^ to 27^th^, and 1.73 at the 28^th^ to 32^nd^ gestational week indicated a high risk of stillbirth, premature pregnancy termination, and a birth weight <2,500 g, with sensitivities of 94.4% and 93.1% as well as specifities of 95.2% and 90.1%, respectively.

## Additional Information

**How to cite this article**: Shen, G. *et al*. Ultrasound prediction of abnormal infant development in hypertensive pregnant women in the second and third trimester. *Sci. Rep.* **7**, 40429; doi: 10.1038/srep40429 (2017).

**Publisher's note:** Springer Nature remains neutral with regard to jurisdictional claims in published maps and institutional affiliations.

## Figures and Tables

**Table 1:** Basic characteristics of the enrolled patients.

	HP group (n = 988)	HypP group (n = 30)	HRHypP (n = 36)	*P*-value
Maternal age (yr)	28.0 ± 3.0	30.5 ± 4.4***	31.9 ± 5.2***^,▴▴▴^	<0.0001
Pre-gestational BMI (kg/m2)	21 ± 2	21.2 ± 2.1	20.8 ± 2.2	0.7214
Primiparous women	84.5% (835/988)	83.3% (25/30)	86.1% (31/36)	0.9958
Current smoker	0	0	0	1.0000
Previous history of PE	0	6.7% (2/30)***	11.1% (4/36)***	<0.0001
Fetal birth weight (g)	3335 ± 327	3336 ± 489	1586 ± 477***^,▴▴▴^	<0.0001
Fetal birth length (cm)	50 ± 1	50 ± 1	36 ± 6.2***^,▴▴▴^	<0.0001
Preterm birth	0 (0/988)	10% (3/30)***	86.4% (19/22)***^,▴▴▴^	<0.0001
IUGR (%)	0	0	31.8% (7/22)***^,▴▴▴^	<0.0001
Caesarean section	36% (356/988)	50% (15/30)	63.6% (23/36)	0.0694
Mean arterial BP (mmHg)	100 ± 10/72	154 ± 12/100 ± 10***	162 ± 22/105 ± 10***^,▴▴^	<0.0001
History of hypertension before pregnancy	0	16.7% (5/30)***	16.7% (6/36)***	<0.0001

Note: Out of 36 cases in the third group, 8 involved fetus death and 6 were aborted fetuses. Therefore, a total of 22 cases were included to calculate the fetal birth weight, premature birth and the IUGR rate. ***P < 0.000 compared to the HP group; ^▴▴▴^*P* < 0.001, ^▴▴^*P* < 0.01 compared to the HypP group.

**Table 2:** Changes of umbilical artery and vein as well as the uterine artery Doppler ultrasound pattern in the three different pregnancy groups at 20–27 weeks of gestation.

Parameter	HP group	HypP group	HRHypP	ANOVA	
	(n = 988)	(n = 30)	(n = 36)	F value	*P*-value
Umbilical artery and vein					
Umbilical artery S/D	3.28 ± 0.60^a^	3.19 ± 0.53^a^	3.88 ± 0.81^b^	19.24	<0.001
Umbilical artery PI	1.11 ± 0.16^a^	1.10 ± 0.16^a^	1.23 ± 0.18^b^	12.30	<0.001
Umbilical artery RI	0.68 ± 0.06^a^	0.67 ± 0.05^a^	0.73 ± 0.05^b^	15.81	<0.001
Umbilical vein flow velocity	14.19 ± 2.59^a^	14.00 ± 3.71^a^	12.08 ± 1.72^b^	11.11	<0.001
Uterine arteries					
Right S/D	2.00 ± 0.45^a^	1.97 ± 0.32^a^	3.62 ± 0.93^b^	206.58	<0.001
Right PI	0.74 ± 0.24^a^	0.72 ± 0.19^a^	1.53 ± 0.39^b^	180.99	<0.001
Right RI	0.48 ± 0.09^a^	0.48 ± 0.08^a^	0.71 ± 0.08^b^	105.96	<0.001
Left S/D	2.10 ± 0.45^a^	2.29 ± 0.89^a^	3.43 ± 0.79^b^	130.20	<0.001
Left PI	0.80 ± 0.23^a^	0.87 ± 0.33^a^	1.42 ± 0.30^b^	117.27	<0.001
Left RI	0.51 ± 0.08^a^	0.53 ± 0.10^a^	0.69 ± 0.07^b^	82.12	<0.001
Left + right PI	1.54 ± 0.4^a^	1.59 ± 0.41^a^	2.95 ± 0.50^b^	207.57	<0.001
Left + right RI	0.99 ± 0.15^a^	1.00 ± 0.15^a^	1.40 ± 0.11^b^	130.77	<0.001
Left + right S/D	4.11 ± 0.76^a^	4.25 ± 0.96^a^	7.05 ± 1.27^b^	242.65	<0.001

Note: The small letters labeling each group denote that the difference between the two groups was statistically significant (SNK method).

**Table 3:** Changes of umbilical artery and vein as well as uterine artery Doppler ultrasound patterns in the three different pregnancy groups at 28–32 weeks of gestation.

Parameter	HP group	HypP group	HRHypP	ANOVA	
	(n = 988)	(n = 30)	(n = 28)	F-value	*P*-value
Umbilical artery and vein					
Umbilical artery S/D	2.65 ± 0.49^a^	2.45 ± 0.39^a^	3.47 ± 0.77^b^	31.87	<0.001
Umbilical artery PI	0.92 ± 0.15^a^	0.86 ± 0.14^a^	1.16 ± 0.17^b^	29.96	<0.001
Umbilical artery RI	0.61 ± 0.07^a^	0.58 ± 0.06^a^	0.70 ± 0.06^b^	22.30	<0.001
Umbilical vein flow velocity	15.95 ± 3.41^a^	16.25 ± 3.32^a^	13.7 ± 2.67^b^	6.24	<0.002
Uterine arteries					
Right S/D	1.86 ± 0.46^a^	1.85 ± 0.34^a^	3.52 ± 1.87^b^	76.26	<0.001
Right PI	0.65 ± 0.22^a^	0.66 ± 0.23^a^	1.39 ± 0.55^b^	101.69	<0.001
Right RI	0.44 ± 0.09^a^	0.44 ± 0.09^a^	0.67 ± 0.12^b^	76.59	<0.001
Left S/D	1.87 ± 0.36^a^	1.91 ± 0.39^a^	3.39 ± 1.04^b^	143.80	<0.001
Left PI	0.66 ± 0.19^a^	0.68 ± 0.24^a^	1.43 ± 0.41^b^	155.37	<0.001
Left RI	0.45 ± 0.08^a^	0.46 ± 0.10^a^	0.68 ± 0.10^b^	85.74	<0.001
Left + right PI	1.32 ± 0.35^a^	1.34 ± 0.43^a^	2.82 ± 0.77^b^	177.21	<0.001
Left + right RI	0.90 ± 0.14^a^	0.90 ± 0.17^a^	1.34 ± 0.19^b^	114.88	<0.001
Left + right S/D	3.73 ± 0.69^a^	3.75 ± 0.66^a^	6.91 ± 2.48^b^	141.63	<0.001

Note: The small letters labeling each group denote that the difference between the two groups was statistically significant (SNK method).

**Table 4:** Sensitivity and specificity of the ultrasound prediction index from the 20^th^–32^nd^ week of gestation.

Effective factors	Cut-off value	AUC (95% CI)	Sensitivity (95% CI)	Specificity (95% CI)	Positive likelihood (95% CI)	Negative likelihood (95% CI)
**Uterine arteries**						
20^th^−27^th^ week						
Left RI	0.55	0.945 (0.929–0.958)	100.0 (90.3–100.0)	72.8 (69.8–75.5)	3.9 (3.4–4.5)	0.11 (0.04–0.30)
Right RI	0.60	0.958 (0.943–0.969)	91.7 (77.5–98.2)	90.3 (88.2–92.0)	9.4 (7.6–11.7)	0.092 (0.03–0.30)
Left + right RI	1.26	0.976 (0.964–0.984)	88.9 (73.9–96.9)	94.5 (92.8–95.8)	16.0 (12.1–21.3)	0.12 (0.05–0.30)
Left PI	1.05	0.947 (0.932–0.960)	88.9 (73.9–96.9)	85.2 (82.8–87.4)	6.0 (5.0–7.3)	0.13 (0.05–0.30)
Right PI	1.01	0.960 (0.946–0.972)	94.4 (81.3–99.3)	89.6 (87.5–91.5)	9.1 (7.4–11.1)	0.062 (0.02–0.20)
Left + right PI	2.35	0.976 (0.965–0.985)	94.4 (81.3–99.3)	95.2 (93.6–96.4)	19.5 (14.6–26.1)	0.058 (0.02–0.20)
28^th^–32^nd^ week						
Left RI	0.57	0.942 (0.910–0.965)	86.2 (68.3–96.1)	91.1 (87.2–94.2)	9.7 (6.5–14.5)	0.15 (0.06–0.4)
Right RI	0.52	0.927 (0.892–0.953)	89.7 (72.6–97.8)	86.2 (81.6–90.0)	6.5 (4.7–8.9)	0.12 (0.04–0.4)
Left + right RI	1.07	0.948 (0.918–0.970)	93.1 (77.2–99.2)	90.4 (86.4–93.6)	9.7 (6.7–14.1)	0.076 (0.02–0.3)
Left PI	0.87	0.953 (0.923–0.973)	93.1 (77.2–99.2)	87.6 (83.2–91.2)	7.5 (5.4–10.4)	0.079 (0.02–0.3)
Right PI	0.97	0.930 (0.895–0.955)	82.8 (64.2–94.2)	92.9 (89.3–95.6)	11.7 (7.4–18.4)	0.19 (0.08–0.4)
Left + right PI	1.73	0.953 (0.923–0.974)	93.1 (77.2–99.2)	90.1 (86.0–93.3)	9.4 (6.5–13.5)	0.077 (0.02–0.3)

Note: The positive likelihood ratio (+LR) has been calculated as screening results of the ratio of the real positive rate and false positive rate. Otherwise, negative likelihood ratio (−LR) means the screening results of the ratio of the real negative rate and false negative rates. The smaller than 1 the −LR data and the bigger than 1 the +LR data are the better measures of accuracy.

**Table 5:** Inter observer reliability and intra-class correlation coefficients (ICC).

	Inter observer ICC	Intra observer ICC
Vmax of umbilical vein	0.89 (0.81–0.94)	0.72 (0.49–0.86)
PI of umbilical artery	0.93 (0.88–0.96)	0.78 (0.57–0.89)
RI of umbilical artery	0.93 (0.88–0.96)	0.76 (0.52–0.88)
PI of right uterine artery	0.97 (0.95–0.98)	0.92 (0.84–0.96)
RI of right uterine artery	0.97 (0.95–0.98)	0.90 (0.80–0.95)
PI of left uterine artery	0.88 (0.79–0.93)	0.78 (0.56–0.89)
RI of left uterine artery	0.84 (0.72–0.91)	0.79 (0.57–0.79)

Note: values above 0.7 indicate a sufficient consistency of the data.

**Table 6:** Doppler ultrasound guideline of uterine artery values for detection of high-risk hypertension pregnancies.

Pregnancy weeks	HP		HRHypP		*P*-value
	Left + right PI	Mean PI	Left + right PI	Mean PI	
20–27	1.54 ± 0.4	0.77 ± 0.23	2.95 ± 0.50	1.48 ± 0.35	<0.001
28–32	1.32 ± 0.35	0.66 ± 0.21	2.82 ± 0.77	1.41 ± 0.48	<0.001

HP: healthy pregnancy; HRHypP high-risk hypertension pregnancy.
